# Editorial: Computational Identification of ceRNA Regulation

**DOI:** 10.3389/fmolb.2022.937505

**Published:** 2022-08-04

**Authors:** Junpeng Zhang, Yun Zheng, Juan Xu

**Affiliations:** ^1^ School of Engineering, Dali University, Dali, China; ^2^ State Key Laboratory of Primate Biomedical Research, Institute of Primate Translational Medicine, Kunming University of Science and Technology, Kunming, China; ^3^ College of Bioinformatics Science and Technology, Harbin Medical University, Harbin, China

**Keywords:** non-coding RNA, miRNA sponge, ceRNA regulation, computational methods, human complex diseases

In molecular biology, gene regulation is a fundamental biological process essential to organisms. Generally, there are two broad levels of gene regulation: transcriptional and post-transcriptional control. In gene regulation, the competing endogenous RNA (ceRNA) regulation (Salmena et al., 2011) mediated by microRNAs (miRNAs) is one of the most commonly studied mechanisms. At both transcriptional and post-transcriptional levels, ceRNA regulation has been shown to be involved in many biological processes, including the initiation and progression of human cancers (Tay et al., 2014). As a novel layer of gene regulation, ceRNA regulation is higher than miRNA regulation in terms of breadth, precision, and complexity (Smillie et al., 2018). Of the many types of ceRNAs, the four most widely investigated are long non-coding RNAs (lncRNAs), pseudogenes, circular RNAs (circRNAs), and messenger RNAs (mRNAs). Heretofore, numerous studies (Tay et al., 2014; Qi et al., 2015; Wang et al., 2016; Misir et al., 2020) have revealed that ceRNAs can act as potential diagnostic biomarkers in clinical applications.

In terms of cost, efficiency, and time consumption, computational methods are useful to guide biological experiments in many areas of biology, and help us derive novel biological insights (Lloyd, 2000; Editors of Nature Methods, 2021). With regard to ceRNA regulation, computational methods have been demonstrated to greatly reduce the time and cost of biological experiments (Le et al., 2017; Li et al., 2018; List et al., 2019; Zhang et al., 2022). Novel computational methods or tools are being presented to shortlist high-confidence ceRNAs for subsequent biological experiments. It is expected that the development of computational methods or tools will drive novel biological insights into the study of ceRNA regulation, and further speed up the research on ceRNA (Figure 1).

**FIGURE 1:**
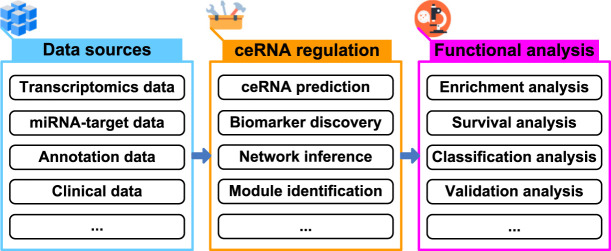
Schema of exploring ceRNA regulation from multiple data sources.

This Research Topic of Frontiers in Molecular Biosciences features a collection of Research articles on the computational or *in silico* identification of ceRNA regulation. It is anticipated that this Research Topic will motivate researchers in the field to accelerate their research on ceRNA and attempt to assist in subsequent experimental design. Sabaie et al. applied a Positive Correlation (PC) method (Zhou et al., 2014; Xu et al., 2015) to investigate the role of lncRNA-related ceRNAs in Autism Spectrum Disorder (ASD), and found that four potential ceRNA axes (*LINC00472*/*hsa-miR-221-3p*/*PTPN11*, ANP32A-IT1/*hsa-miR-182-5p*/*S100A2*, *LINC00472*/*hsa-miR-132-3p*/*S100A2,* and *RBM26-AS1*/*hsa-miR-182-5p*/*S100A2*) may be involved in ASD pathogenesis. To understand the potential prognostic and immunological roles of *CCNA2* in pan-cancer, Chen et al. performed a pan-cancer analysis to identify the upstream regulatory networks of *CCNA2* and *CCNA2*-related ceRNAs in 33 tumor types. Moreover, Guo et al. systematically analyzed and integrated chromosomal instability-related dysregulated ceRNAs characteristics in lung adenocarcinoma (LUAD), and discovered that the identified 12 dysregulated ceRNAs (*AMOTL1*, *EFNB2*, *FGF2*, *FURIN*, *CCND2*, *IFNG*, *ITGB4*, *RHOV*, *LINC00473*, *LINC00707*, *MIR497HG*, and *RP11-16E12.2*) are closely associated with multiple cancer progresses, especially immune-related pathways. In addition, by integrating widely used computational methods and several public databases, Song et al. developed an interactive R/Shiny tool, ceRNAshiny, for identification and analysis of ceRNA regulation. Overall, these studies applied existing methods or developed new tools to identify ceRNA regulation from bulk transcriptomics data, which provided potential ceRNAs for subsequent biological experiments.

The explosive growth of biological data, especially omics data, provides opportunities for computational biologists or bioinformaticians to develop methods or tools to unearth biological implications hidden in the abundant data. Recently, although heterogeneous data (e.g., omics and non-omics data) has opened a way to explore ceRNA regulation, how to effectively integrate multiple data sources when developing novel computational methods is still a challenge. Moreover, the identification of ceRNA regulation is generally a computation-intensive task. For the fast inference of ceRNA regulation in large-scale data, it is necessary to develop methods or tools with parallel computing. Until now, existing computational methods are only confined to the study of ceRNA regulation at the multi-sample level, rather than the ceRNA regulation at the single-sample level. This may not precisely solve the heterogeneity of ceRNA regulation across individual samples. Additionally, with the development and innovation of single-cell and spatial sequencing technology, it will be an exciting direction to develop novel methods or tools for exploring ceRNA regulation at the single-sample level. Finally, it is extremely important to link ceRNA regulation with biological functions. However, how to connect predicted ceRNA regulation with biological functions (e.g., human diseases) and establish feasible benchmarks or guidelines for analyzing ceRNA regulation is still a challenge. Altogether, to identify ceRNA regulation for assisting in subsequent experimental design and discover potential ceRNA biomarkers for clinical application, developing practical methods or tools is indispensable to the investigation of ceRNA regulation.

## References

[B1] Editors of Nature Methods (2021). Computation and biology: A partnership. Nat. Methods 18, 695. 10.1038/s41592-021-01215-2 34239101

[B2] Le T. D. Zhang J. Liu L. Li J. (2017). Computational methods for identifying miRNA sponge interactions. Brief. Bioinform. 18, 577–590. 10.1093/bib/bbw042 27273287

[B3] Li Y. Huo C. Lin X. Xu J. (2018). Computational identification of cross-talking ceRNAs. Adv. Exp. Med. Biol. 1094, 97–108. 10.1007/978-981-13-0719-5_10 30191491

[B4] List M. Dehghani Amirabad A. Kostka D. Schulz M. H. (2019). Large-scale inference of competing endogenous RNA networks with sparse partial correlation. Bioinformatics 35, i596–i604. 10.1093/bioinformatics/btz314 31510670 PMC6612827

[B5] Lloyd A. (2000). Computational methods in molecular biology. Briefings Bioinforma. 1, 315–316. 10.1093/bib/1.3.315

[B6] Misir S. Hepokur C. Aliyazicioglu Y. Enguita F. J. (2020). Circular RNAs serve as miRNA sponges in breast cancer. Breast Cancer 27, 1048–1057. 10.1007/s12282-020-01140-w 32715419

[B7] Qi X. Zhang D.-H. Wu N. Xiao J.-H. Wang X. Ma W. (2015). ceRNA in cancer: possible functions and clinical implications. J. Med. Genet. 52, 710–718. 10.1136/jmedgenet-2015-103334 26358722

[B8] Salmena L. Poliseno L. Tay Y. Kats L. Pandolfi P. P. (2011). A ceRNA hypothesis: The rosetta stone of a hidden RNA language? Cell 146, 353–358. 10.1016/j.cell.2011.07.014 21802130 PMC3235919

[B9] Smillie C. L. Sirey T. Ponting C. P. (2018). Complexities of post-transcriptional regulation and the modeling of ceRNA crosstalk. Crit. Rev. Biochem. Mol. Biol. 53, 231–245. 10.1080/10409238.2018.1447542 29569941 PMC5935048

[B10] Tay Y. Rinn J. Pandolfi P. P. (2014). The multilayered complexity of ceRNA crosstalk and competition. Nature 505, 344–352. 10.1038/nature12986 24429633 PMC4113481

[B11] Wang Y. Hou J. He D. Sun M. Zhang P. Yu Y. (2016). The emerging function and mechanism of ceRNAs in cancer. Trends Genet. 32, 211–224. 10.1016/j.tig.2016.02.001 26922301 PMC4805481

[B12] Xu J. Li Y. Lu J. Pan T. Ding N. Wang Z. (2015). The mRNA related ceRNA-ceRNA landscape and significance across 20 major cancer types. Nucleic Acids Res. 43, 8169–8182. 10.1093/nar/gkv853 26304537 PMC4787795

[B13] Zhang J. Liu L. Xu T. Zhang W. Li J. Rao N. (2022). Time to infer miRNA sponge modules. Wiley Interdiscip. Rev. RNA 13, e1686. 10.1002/wrna.1686 34342388

[B14] Zhou X. Liu J. Wang W. (2014). Construction and investigation of breast-cancer-specific ceRNA network based on the mRNA and miRNA expression data. IET Syst. Biol. 8, 96–103. 10.1049/iet-syb.2013.0025 25014376 PMC8687191

